# Quality of Life, Psychological Interventions and Treatment Outcome in Tuberculosis Patients: The Indian Scenario

**DOI:** 10.3389/fpsyg.2016.01664

**Published:** 2016-10-27

**Authors:** Vidyullatha Peddireddy

**Affiliations:** Department of Biotechnology and Bioinformatics, University of HyderabadHyderabad, India

**Keywords:** psychological interventions, quality of life, tuberculosis, treatment outcome, India, psychological distress

## Abstract

**Objective:** Psychological distress is being recognized in individuals affected with many diseases since it affects quality of life (QOF) and has gained importance in the clinical settings. Psychological interventions and their effect on the treatment outcome have yielded encouraging results in many diseased conditions. Tuberculosis (TB) ranks as a deadly disease resulting in millions of deaths worldwide. However, the effect of TB on the psychological status of patients and interventions to improve treatment outcome is neglected, especially in underdeveloped and developing countries.

**Methods:** Systematic review of research papers that published on the QOF in TB and the effect of psychological interventions on treatment outcome were conducted.

**Results:** Tuberculosis patients experience high levels of stress and decreased QOF. In the Indian scenario, TB patients undergo immense psychological stress similar to what is reported in other locations. Psychological interventions renewed hope on life and adherence to medication and treatment outcomes. Such psychological interventions are not practiced in Indian clinical settings.

**Conclusion:** There is an urgent need for both governmental and non-governmental organizations to devise strategies to include psychological interventions mandatory during TB treatments. In the absence of such interventions, the fight against TB in India will remain incomplete.

## Introduction

Psychological distress is a common phenomenon experienced by humans in a variety of conditions, but is vaguely understood. It is defined as a state of mind where the emotional suffering is associated with depression and anxiety (Drapeau et al., 2011; Peddireddy, 2016). Psychological distress due to many living conditions such as work pressure, societal changes, financial needs and changing life style leads to diseased states and the mortality in these individuals is on the rise (Kessler et al., 2009). It is projected that individuals who experience immense psychological distress have less survival rates compared to the general population (NASMHPD, 2006). On the other hand, people affected with any disease also undergo psychological distress and the medical outcome in these individuals is dependent on the psychological interventions (Trangle et al., 2016). The necessity of psychological interventions during diseased conditions is important due to the fact that individuals with chronic diseases exhibit altered mental status resulting in risky behaviors such as non-adherence to treatment, altered life style (tobacco smoking, alcoholism) and indulging in unsafe sexual practices (Pasco et al., 2008; Huther et al., 2013; Moore and Posada, 2013; Pachi et al., 2013; Behera et al., 2014; Pezzoni and Kouimtsidis, 2015). The risky behavior stems from the feeling that they will not survive for long and all the pleasures of life should be experienced as soon as possible. Because of the risky behavior resulting from psychological distress, these individuals are likely to die 25 years earlier than the normal population (NASMHPD, 2006). In addition to this, psychological distress induced inability to take care of their own health affects quality of life (QOF) in terms of physical inability and the extent of suffering from chronic pain (Veggi et al., 2004).

A variety of diseases such as cancer, diabetes, hypertension, AIDS, malaria and tuberculosis (TB) affect millions of people around the world. The incidence and the extent to which the QOF is affected in these diseased conditions depend on the status of the disease, geographical location, medical interventions and most importantly the psychological interventions. Though some of the diseases are not common in the developed world, TB remains a major disease entity causing millions of deaths annually across the world, with higher rates of mortality in the developing nations (WHO, 2015a). The treatment for TB depends on the drug resistant nature of the causative agent, i.e., *Mycobacterium tuberculosis* (*M.tb*). Most *M.tb* strains are sensitive to the first line drugs such as isoniazid (INH) and rifampin (RIF) causing active TB and the disease is completely treatable in about 6 months. Multi drug resistant tuberculosis (MDR-TB) is caused by the *M.tb* strains that are resistant to first line drugs and the treatment period is up to 2 years. Mismanagement of MDR-TB patients resulted in the emergence of *M.tb* strains that cause Extreme drug resistant tuberculosis (XDR-TB) which need treatment with second line of drugs such as amikacin and kanamycin, or capreomycin. It is very surprising to note that the number of countries that reported confirmed cases of XDR-TB has increased from 49 to 84 in the years between 2008 and 2013 (WHO, 2013). Treatment of MDR-TB and XDR-TB are very complicated due to logistic difficulties in acquiring the first and second line drugs, requirement for frequent administration by intravenous route, requirement of a surgery, side effects of these drugs, strict adherence of the patient and the long duration of treatment and higher mortality rate (Iseman, 1993; Ormerod, 2007). Further, social factors such as financial implications, loss of employment and social stigma are also associated (Booker, 1996). An important aspect of *M.tb* is its ability to remain latent in the host for extended period of time which ranges from months to years. A person with latent TB appears normal, but can exhibit symptoms when the causative agent gets reactivated due to favorable survival conditions, such as waning of the immune system. Patients experiencing re-emergence of TB after being latent are caught unaware and experience a sudden change in life and the associated implications. Another important feature of TB is its association with HIV-infected patients. Because of the unique life cycle and survival strategies of *M.tb*, TB remains a global challenge throughout the world. Because of its complexity involved in TB and the associated clinical and social implications, people affected with this disease are subjected to enormous psychological distress regardless of the type of TB they are affected with.

In the present day world, outstanding research contributions have advanced the strategies to treat TB targeting the pathogen and the host at the molecular level. I am interested in studying the contribution of mycobacterial proteins during latency and the immune pathways that are targeted by *M.tb* to establish a successful latent phase in the host (Peddireddy et al., 2016). My studies also include interaction with TB patients to record the disease status. General conversations with them made me to realize that they undergo immense stress and have no support system to cope up with the psychological trauma. A thorough revision of studies conducted throughout the world suggested that, at the patient level, psychological interventions seems to make a lot of difference in the morbidity and mortality. In the advanced nations, psychological stress experienced by TB patients and the interventions made to address them have enhanced the treatment quality, morbidity and mortality in all types of TB. Nature of psychological distress in Indian TB patients still remains an unexplored area, though this country is ranked among the high burden countries. Further, India is one of the fastest growing economies and the quality of medical treatment has increased leaps and bounds. However, treatment of TB patients in combination with psychological interventions is not being practiced by the medical professionals. It is a pity to note that such psychological interventions are not in the reach of Indian TB patients. In light of the above mentioned reasons, an attempt is made in this review to draw the attention of health care providers toward the urgent need to include psychological interventions for the treatment of this disease in the Indian scenario.

## Methods

Direct interaction with TB patients and extensive literature review was conducted. Studies that relate to the QOF in TB patients and the effect of psychological interventions on the treatment outcome were identified using PubMed search with the keywords: TB and QOF; TB and psychological intervention; TB and treatment outcome and psychological aspects.

## Observations

### Psychological Distress in Tuberculosis Patients

Psychological evaluation in many clinical conditions including TB has gained importance in the recent years. Based on a number of studies it is widely believed that psychological distress is evident in TB patients throughout the world. Depression and anxiety rates that contribute to psychological stress were found to be high in TB patients compared to the general population (Shen et al., 2014) and this trend is more evident in the developing and under developed nations such as Nigeria, Ethiopia, South Africa, and Pakistan (Aamir and Aisha, 2010; Coker et al., 2011; Peltzer et al., 2012b). It is interesting to note a positive correlation between adverse life events with increased incidence of TB (Geldenhuys et al., 2011). Further, the morbidity in TB was found to be associated with psychological distress (Doherty et al., 2013). Besides clinical manifestations, social and the individual’s behavioral aspects contribute to the high psychological distress in TB patients. These factors include social status, poverty, education, perception toward the disease and the anti-TB medications, gender, support from the family and friends, marital status, smoking and alcohol consumption (Peltzer et al., 2012a,b; Pachi et al., 2013; Masumoto et al., 2014; Peltzer and Louw, 2014; Feng and Xu, 2015).

### Quality of Life in Tuberculosis Patients

For many decades, it is known that TB inflicts physical suffering and the analysis of this infection on the health related quality of life (HRQOL) has emerged to be an active area of investigation in the recent years. In general terms, QOL is defined as the physical, emotional and social well-being status during a disease as perceived by the patient (Roila and Cortesi, 2001). However, these definitions change depending on the type of disease the patient is experiencing. In the context of TB, because of the complexity displayed by *M.tb*, an individual may be infected (latent) or carrying a disease (active). Further, the disease can be pulmonary or extra pulmonary infecting many organs. Thus a specific parameter that defines the HRQOL in this diseased condition is not possible. Though QOF assessment was standardized and practiced in diseases such as cancer, the attention paid to assess the QOF in TB patients is meager. Until 2004, only 60 studies were retrieved that addressed the QOF in TB patients (Chang et al., 2004). A number of generic instruments were used to measure HRQOL in TB, though none of them were specifically designed for this disease (**Table 1**). A major stumbling block to measure HRQOL in TB patients is the nature of controls to be used. Since TB is generally associated with lower socio-economic groups or specific ethnic groups, comparing them with the general population does not reflect the actual status. The best possible control would be the population having latent infection, though these individuals themselves have impaired HRQOL when compared to healthy controls (Schwindenhammer et al., 2013).

**Table 1:** Salient features of instruments used to measure HRQOL in TB patients.

Name of the instrument	Number of items	Remarks
**Generic**		
Brief Disability Questionnaire (BDQ)	11	HRQOL inversely proportional to score
Duke Health Profile (DUKE)	63	Higher the score, better is the HRQOL
Dysfunctional Analysis Questionnaire (DAQ)	50	HRQOL inversely proportional to score
Euro-QoL (EQ 5D)	5	Higher the score, better is the HRQOL
General Health Questionnaire 12 (GHQ 12)	60	Higher the score, better is the HRQOL
Health Utilities Index (HUI)	8	Score 0 indicates death and 1 indicates perfect health
Life Satisfaction Index Z	13	Higher the score, better is the HRQOL
Patient Health Questionnaire – 9 (PHQ9)	9	Higher the score, worse is the HRQOL
Severe Respiratory Insufficiency Questionnaire (SRI)	49	Higher the score, better is the HRQOL
Symptoms Check List – 90 (SCL-90)	90	Higher is the score, worse is the HRQOL
Sheehan Disability Scale (SDS)	20	Higher is the score, worse is the HRQOL
SF-6D utility score	11	Higher the score, better is the HRQOL
SF-36 Health Survey (SF-36)	36	Higher the score, better is the HRQOL
Standard Gamble	1	Higher the score, better is the HRQOL
Visual Analog Scale (VAS)	10	Score 0 indicates death and 10 indicates perfect health
World Health Organization’s Quality of Life–BREF (WHOQOL-BREF)	26	Higher the score, better is the HRQOL
**Anxiety – Depression**		
Beck Depression Inventory (BDI)	21	>13 score indicates depression
Beck Depression Inventory (BDI Short Form)	13	>16 score indicates depression
Centre for Epidemiologic Studies Depression Scale (CES-D)	15	Higher score indicates depression
Hospital Anxiety and Depression Scale (HAD)	14	Higher scores indicate depression and anxiety
Kessler 10	10	Higher the score, better is the HRQOL
Mood Adjective Check List Short Form (MACL)	38	Evaluates calmness, pleasantness and activation
Rosenberg Self-Esteem Scale (RSE)	10	Higher the score, better is the HRQOL
Self-Rating Anxiety Scale (SAS)	20	Higher is the score, worse is the HRQOL
State-Trait Anxiety Inventory Short Form (STAI-6)	6	Higher is the score, higher is the anxiety
**Disease specific**		
Schedules for Clinical Assessment in Neuropsychiatry	1872	Higher is the score, worse is the HRQOL
St. George Respiratory Questionnaire Short Form (SGRQ)	50	Evaluates symptoms, activity and impact
DR-12	12	TB-specific; Higher the score, better is the HRQOL
World Health Organization’s Quality of Life – HIV (WHOQOL-HIV)	100	HIV specific; Higher the score, better is the HRQOL
MOS-HIV	35	HIV specific

The QOF in active TB patients was reported by many studies and the objectives were widely varied. Holistic HRQOL, psychological morbidity and physical well-being were some of the parameters on which the studies focused. Nevertheless, it is generally observed that lower levels of QOF and physical pain were the most common outcomes of these studies (Brown et al., 2015). Some studies that focused on the QOF in active TB patients in comparison with latent TB revealed a lower health status with a Physical Component Survey score of 44.8 and Mental Component Survey score of 54.7 in the active TB patients. The corresponding PCS and MCS scores in the latent TB patients were 54.7 and 50.3 respectively (Guo et al., 2008). Qualitative research methodologies provided the reasons for reduced QOF in TB patients. Upon diagnosis, TB patients experience stigmatization and social isolation, loss of employment and inability to access higher levels of health care. On the other hand, hospitalization as one for the treatment strategies had profound effect on the QOF in TB patients. Those receiving supervised care in a hospital setting is always recommended and is predicted to have better QOF than those compared to those receiving ambulatory care (Diel et al., 2012). Assessment of QOF post TB treatment is understudied. However, social stigma and its repercussions affected the QOF in individuals who have been completely treated with TB (Dias et al., 2013). Further, the St. Georges Respiratory Questionnaire (SGRQ) scores were found to be increased (worse health status) in subjects who underwent at least 20 weeks of TB treatment compared to those having latent TB (Pasipanodya et al., 2007). Assessment of QOF did not receive much attention in patients with extra-pulmonary TB. A decline in QOF was reported in patients with extra-pulmonary TB (Dhingra and Rajpal, 2005) and on the contrary no significant differences in were observed in a Chinese study (Chamla, 2004). The lack of assessment of QOF in patients with extra-pulmonary TB underlines the urgent need to take up such studies since this form of the disease also affects a large number of people around the world.

Co-infection of HIV in TB patients is very common (WHO, 2015b) and are subjected to high level of stigmatization (Hsiung et al., 2011) and very low QOF. Exclusion of TB patients with HIV co-infection or not declaring the status of HIV infection and the associated antiretroviral therapy in the study subjects in many studies resulted in poor understanding of the levels of QOF in this subset of patients. In a large South African study comprising of 4900 participants, significantly lower health status and QOF were observed in HIV infected TB patients than their TB alone infected counterparts (Louw et al., 2012; Peltzer et al., 2013). The physical health summary scores were found to be significantly different in TB patients with or without HIV co-infection (Dowdy et al., 2013). In an Ugandan study, the visual analog scale (VAS) score was significantly improved in patients receiving TB treatment over a period of 6 months. But the scores were not significant between the HIV-TB co-infected and TB along infected groups (Babikako et al., 2010, 2011). The QOF in HIV-TB co-infected and TB alone infected patients was significantly improved after a 6 months anti TB therapy (Deribew et al., 2013). However, the HIV alone infected counter parts did not show much improvement in the QOF since they were recruited from clinics that were administering antiretroviral therapy for a long time (Deribew et al., 2009). HIV patients receive antiretroviral therapy immediately upon diagnosis, and thus assessing QOF in HIV-TB infected patients who are not receiving antiretroviral drugs becomes impossible. Since a direct comparison of QOF in HIV-TB infected patients with or without antiretroviral therapy, but receiving only anti-TB therapy cannot be reported, the interference of antiretroviral therapy has always been a matter of concern in these studies.

Another complexity that is encountered during TB treatment is drug resistance and it is anticipated that this condition results in severe impairment of QOF since the patient has to be under medical isolation leading to loss of employment and social activities. The QOF seems to be higher in MDR-TB compared to XDR-TB patients (Conradie et al., 2014), suggesting that the severity of the disease has a direct effect on the well being. Because of the use of highly powerful antibiotics in different combinations for extended periods of time, MDR-/XDR-TB, neurological disorders, hearing loss and reduced body weight are evident, which has a profound effect on the QOF (Seddon et al., 2012, 2013; Bassili et al., 2013; Melchionda et al., 2013). The global distribution of MDR-/XDR-TB varies highly between geographical regions (WHO, 2015b) and the importance given for assessing QOF also varies globally. Thus it becomes very challenging to suggest a common level of loss of QOF in TB patients. Because of high morbidity and mortality in MDR-/XDR-TB patients, not many studies were conducted to assess the quality of living in these patients. On a severe note, the survival rate of patients with MDR-/XDR-TB and HIV co-infection reduces drastically and this makes it very difficult to assess QOF. A Brazilian study on MDR-/XDR-TB indicated lower Airways Questionnaire 20 (AQ20) scores (improvement in health symptoms) in terms of lung function tests, chest radiographs and walk tests (Godoy et al., 2012).

An interesting feature of *M.tb* infection is its ability to remain latent in the host and cause asymptomatic TB. It is believed that identifying individuals with latent TB is a significant measure to reduce the global burden of TB and to evolve better strategies for the treatment. Labeling an individual as a latent TB patient causes distress and anxiety and thus resulting in loss of QOF. A significant reduction in HRQOL was observed in individuals with latent TB than their healthy counterparts (Bauer et al., 2002). Factors that contribute to the decreased QOF in latent TB patients is anxiety, depression, social stigma, side effects of treatment and unwillingness to believe that they are having disease despite appearing normal and healthy.

Besides adults, children are also at a high risk for TB (WHO, 2015b). Unfortunately, no studies have been conducted that included TB patients aged less than 10 years (Bauer et al., 2013). Other neglected groups that did not receive attention to evaluate the QOF and TB are individuals working in health care settings, prisoners, pregnant mothers and children born to them. Thus, till today, the real picture of QOF in TB patients is not clear.

## The Indian Scenario

In the Indian scenario, assessing QOF in TB patients has been a neglected area for a long time (Aggarwal, 2010). It gained momentum in the recent years only due to increased awareness on the benefits of psychological interventions and measuring of the treatment outcome in terms of the overall health status of the patient. Indian TB patients experience stigma and this is dependent on the social, geographical and gender factors. In the capital city of India, Delhi, immense stigma was experienced by patients and about 60% of the patients concealed it from family members and friends. The stigma was more prevalent in women (Dhingra and Khan, 2010). In a study that recruited TB patients from South India, it was observed that distress and anxiety was observed in about 50% of the study subjects when disclosed about the diagnosis and about 9% of them contemplated suicide (Rajeswari et al., 2005). Social stigma was prevalent and it did not vary between men and women. Despite a significant increase in the health status, only 54% of them perceived “happy mental status” after TB treatment. In a recent study involving TB patients from rural parts of Gujarat, a Northern state of India, psychosocial reaction was severe when informed of the diagnosis for TB and the main worries were mainly about the deadliness of the disease, treatment options, embarrassment, social stigma and the feeling that fate was not kind to them (Thakker et al., 2014). Further, in a rural Andhra Pradesh region of India, it was widely believed that TB is caused by natural and supernatural origins, witchcraft, evil, and imbalance in the quantity of consumption of hot and cold foods and people with TB are considered as those with ill fate (Venkatraju and Prasad, 2010). Severe depressive symptoms were evident in MDR-TB patients, which then improved substantially during the course of treatment (Das et al., 2014). Similarly, psychological impairment in the regions of the brain is associated with family, social and personal activities in TB patients of Delhi region (Bhatia et al., 2000).

The HRQOL was found to be severely impaired in pulmonary TB and substantial improvement was evident after treatment in Indian TB patients (Aggarwal, 2010). The mean scores for QOF were lower in TB patients and the most affected domains were physical and psychological followed by social and environmental (Dhuria et al., 2008, 2009). In another North Indian cohort, it is reported that HRQOL was better in pulmonary TB patients than the MDR-TB patients (Sharma et al., 2014). In both these conditions, besides social stigma, the effect on psychological and environmental domains was more profound than in the physical and social domains.

## Psychological Interventions and their Effect on Tuberculosis Treatment Outcome

As the saying goes, pain is inevitable; suffering is optional, for every disease, suffering either in physical or emotional or psychological form is inevitable. How a patient deals with these depends on the will power and the support extended by family and friends. Despite the invention of advanced technologies to diagnose diseases and development of powerful drugs to cure them, the treatment outcomes vary among patients. The pharmacologic profile of the patient is an important determinant for the treatment outcome. Another crucial aspect that has gained importance to achieve better treatment outcomes in many diseases is the psychological intervention. Such non-medical interventions have been practiced in the developed nations for the treatment of many diseases including TB. The psychological interventions for TB are more complicated when compared to other diseases because of the negative perception and stigma toward this disease in the society on one side and the biological complexities on the other side. Despite the complexities, psychological interventions have improved the prevention and treatment outcome of TB.

Prevention of TB infection also seemed to be psychologically associated. In a Vietnamese population, the high incidence of TB was reduced by developing a culturally sensitive educational and psychological intervention programs (Houston et al., 2002). Stigma experienced by people due to TB contributes to keeping the disease as a secret, avoiding visiting the clinics and the reluctance to comply with the prescribed medication. A literature review of about 170 research articles indicated that stigma toward TB was highly prevalent and contributes to delayed treatment and non-compliance to medications (Courtwright and Turner, 2010). It is surprising to note that not many studies have focused on psychological interventions to reduce stigma and its effect on the treatment outcome. The few studies conducted indicated mixed results. Interventions included creation of TB clubs, TB education/support programs and empowering TB infected individuals (Courtwright and Turner, 2010). Creation of TB clubs had positive effect on reducing TB stigmatization and the treatment outcomes (Demissie et al., 2003; Macq et al., 2008). Effective questionnaires that can measure stigma, assessing the effect of reducing stigma on treatment outcome and psychological interventions to reduce stigma are to be taken up at a large scale throughout the world especially in those regions where the prevalence of TB is very high (Courtwright and Turner, 2010).

One of the major obstacles in treating TB patients is their non-adherence to the treatment regimen and these results in prolonged disease transmission and development of resistance to the anti-TB drugs. Psychological counseling improved the compliance to treatment and the successful treatment of the disease in an Ethiopian cohort (Tola et al., 2016). An extensive review on the effect of psychological interventions on adherence to medication suggested that improvement in compliance toward medication was observed when psychological aspects such as depression were better managed in TB patients (Pachi et al., 2013). The compliance to medication was found to be improved in MDR-TB patients who underwent psychosocial support program (PSS) (Kaliakbarova et al., 2013). A programmatic psychological intervention improved the patients’ treatment compliance, outcome and levels of CD4+T lymphocytes suggesting that besides changing the mental attitude, physiological responses can also be improved due to such interventions (Wei et al., 2016). Psychotherapy in TB patients decreased dysfunctional beliefs and increased the hope to get well soon and lead a happy life with a concomitant compliance to treatment (Safa et al., 2013). Systematic review and meta-analyses indicated implementation of simple psychological interventions to scale up MDR-TB programs for a better treatment outcome (Toczek et al., 2013; Thomas et al., 2016). In the Indian context, direct population based studies, literature survey and meta-analyses indicate psychological effects in TB patients and comprehensive studies that focus on the benefits of psychological intervention on the treatment outcome are not available (Thomas et al., 2016). Compliance to medication was significantly improved in Indian TB patients who underwent multiple psychological sessions (Janmeja et al., 2005). However, the implementation of psychological interventions and its application to TB patients needs to operationalized at different levels (**Table 2**) in this country.

**Table 2:** Operational plan to provide psychological support to tuberculosis patients in the health care system.

Level	Action	Targets to be achieved
National	^•^ Wide publicity in social media on the prevention and management of tuberculosis. ^•^ Publicize the importance of psychological support to tuberculosis patients.	^•^ Awareness among the people to maintain hygiene and avoid transmission of tuberculosis. ^•^ Reduce stigma toward tuberculosis patients and also empowering these patients for proper treatment.
Community	^•^ Campaigning at household and community levels on the prevention, management and support systems (medical and psychological) available for tuberculosis patients.	^•^ Awareness on tuberculosis and knowledge on access to treatment. ^•^ Support tuberculosis patients and guide them toward treatment options.
Hospital	^•^ Implement training programs on psychological interventions and their benefits on treatment regimen. ^•^ Support groups should be created and managed by an experienced counselor or social worker. ^•^ A health worker (nurse) trained in management of TB should be co-opted. ^•^ Creating a support group should be based on a clear eligibility criteria. ^•^ Review of each support group meeting to be made by the counselor and steps initiated for the betterment of the next meeting, based on the experiences in the current meeting.	^•^ Sensitize the medical practitioners on the psychological needs of tuberculosis patients and the beneficial effects of such interventions on recovery. ^•^ Assessment of emotional and socioeconomic issues. ^•^ Establishment of drug abuse, if any and initiate treatment accordingly. ^•^ Identification of patients with depression and prescribe antidepressant regimen. ^•^ Analyze the drugs being given to the patients and the possible psychological side effects. ^•^ Involving family members to provide emotional support.

Health literacy is one of the most important factors that determine the incidence, transmission and morbidity of TB. In general, the incidence of many diseases including TB is more in populations where health literacy levels are lower. Limited literacy on health issues are associated with lower practices of prevention measures, immunization, use of antibiotics, seeking proper medical treatment, especially in infectious diseases such as TB and malaria (Castro-Sanchez et al., 2016; Osborne et al., 2016). Increased morbidity and mortality due to infectious diseases is observed in adults with limited health literacy (Bostock and Steptoe, 2012). The awareness on TB was found to be very low in a tribal group in central India (Muniyandi et al., 2015). Though awareness on TB was good in an Indian urban slum population, information on access to proper medical care was not known (Chinnakali et al., 2013). It is suggested that TB patients with low health related literacy had higher psychological distress (Theron et al., 2015). Thus it appears that health literacy, morbidity due to TB and psychological well-being are interlinked. It is suggested that investments on critical health literacy are crucial and worthwhile to develop qualitative and quantitative approaches of evaluating health literacy and their application to enhance QOF (Chinn, 2011).

## Conclusion

Psychological stress is prevalent in TB patients throughout the world. The importance given to this aspect varied in different geographical locations and it is very surprising to note that studies relevant to this aspect were not conducted on a large scale basis in countries that contribute to the highest global burden of TB. Though few studies focused on TB associated psychological distress and QOF, the benefits of psychological intervention for treatment outcome are not conducted.

It is high time that all the agencies in India that work for reducing TB burden realize that improving health literacy and adopting psychological interventions should be practiced to improve the QOF, treatment outcome and prevention of this disease. Mandatory psychological counseling to patients and training on psychological interventions to medical practitioners should be introduced in all health care units. Since, majority of the TB patients are on long treatment regimen and remaining in hospital is not mandatory, online portals that will allow interaction between the patient and the health care provider should be launched. Telephonic access to psychological practitioners should be made available to patients to express their difficulties and the same should be communicated to the health care providers to design better treatment strategies.

## Author Contributions

VP conducted literature search and compiled the manuscript.

## Conflict of Interest Statement

The author declares that the research was conducted in the absence of any commercial or financial relationships that could be construed as a potential conflict of interest.
